# A Pilot Study of Service Utilisation Pathways of Patients With Distinct Psychotic and Antisocial Typologies

**DOI:** 10.1192/bjo.2022.177

**Published:** 2022-06-20

**Authors:** Alexander Challinor, Neil Meggison, Jonathon Whyler, Phoebe Cresswell, Leah Evans, Michael Bingley, Praveen Somarathne, Jodi Thompson, Dawn Washington, Taj Nathan

**Affiliations:** 1 Mersey Care NHS Foundation Trust, Liverpool, United Kingdom.; 2 University of Liverpool, Liverpool, United Kingdom; 3 Health Education North West, Manchester, United Kingdom; 4 Lancashire and South Cumbria NHS Foundation Trust, Lancashire, United Kingdom; 5 Cheshire and Wirral Partnership NHS Trust, Chester, United Kingdom; 6 University of Chester, Chester, United Kingdom; 7 Liverpool John Moores University, Liverpool, United Kingdom

## Abstract

**Aims:**

There is a developing body of research that suggests that there may be distinct categories of patients that can explain the relationship between psychosis and antisocial behaviours. Specifically, three pathways of offending, antisocial behaviour and psychosis have been described and there is an evolving empirical evidence base to suggest that these pathways are aetiologically distinct. Firstly, there is a pathway for early-start offenders, which have been identified as those with psychosis preceded by Conduct Disorder (SZ + CD). Secondly, a group that start to display antisocial behaviours in parallel to the onset of psychosis (SZ-AS). The third group involves those with a long history of a psychotic disorder and no history of antisocial behaviours, who will present to services following a first conviction for non-violent or violent crime (SZ). The authors hypothesise that each typology will utilise services differently throughout the clinical trajectory. This pilot study aimed to (i) examine the concurrent validity of the antisocial behaviour and psychosis typologies, and (ii) examine differences in the service utilisation patterns of patients between these groups.

**Methods:**

The sample consisted of adult male patients admitted to low and medium secure forensic hospitals within the Northwest of England. A total of 90 patients were used.

A categorisation checklist was developed, and the typology of patients determined from data collected from electronic health records. Data were collected on patient demographics, psychiatric diagnosis, aetiological factors, and service utilisation. Two researchers reviewed the data and determined the typology. Statistical analysis aimed to assess the difference in aetiological variables between the typologies and examine the relationship with how each typology utilised services.

**Results:**

This study provided further evidence of distinguishing characteristics emphasising typology heterogeneity.

The CD-SZ group were more likely to have utilised mental health services <18 years (70%, p = 0.062), and to have used services preceding a diagnosis of psychosis (60%, p = 0.011). Following the onset of a psychotic disorder, the AS-SZ and SZ groups had a higher proportion that used general adult psychiatry services (p = 0.031), with CD-SZ coming in to contact with forensic psychiatry services and criminal justice services earlier and more frequently.

**Conclusion:**

This study demonstrates that each typology has a different clinical trajectory through mental health services. This provides further empirical evidence towards different clinical typologies and trajectories of individuals with psychosis and anti-social behaviour. Understanding more about how these typologies utilise services will enable clinicians to introduce interventions help develop effective management plans that address the distinct characteristics of each typology of offender with psychosis.

